# Can Skin Exposure to Sunlight Prevent Liver Inflammation?

**DOI:** 10.3390/nu7053219

**Published:** 2015-05-05

**Authors:** Shelley Gorman, Lucinda J. Black, Martin Feelisch, Prue H. Hart, Richard Weller

**Affiliations:** 1 Telethon Kids Institute, University of Western Australia, 100 Roberts Rd, Subiaco, Western Australia 6008, Australia; E-Mails: lucinda.black@telethonkids.org.au (L.J.B.); prue.hart@telethonkids.org.au (P.H.H.); 2 Clinical and Experimental Sciences, Faculty of Medicine, University of Southampton, Southampton General Hospital, Southampton, SO16 6YD, UK; E-Mail: M.Feelisch@soton.ac.uk; 3 University of Edinburgh, MRC Centre for Inflammation Research, Edinburgh, EH16 4TJ, UK; E-Mail: richard.weller@ed.ac.uk

**Keywords:** sunlight, vitamin D, nitric oxide, liver, inflammation, non-alcoholic fatty liver disease

## Abstract

Liver inflammation contributes towards the pathology of non-alcoholic fatty liver disease (NAFLD). Here we discuss how skin exposure to sunlight may suppress liver inflammation and the severity of NAFLD. Following exposure to sunlight-derived ultraviolet radiation (UVR), the skin releases anti-inflammatory mediators such as vitamin D and nitric oxide. Animal modeling studies suggest that exposure to UVR can prevent the development of NAFLD. Association studies also support a negative link between circulating 25-hydroxyvitamin D and NAFLD incidence or severity. Clinical trials are in their infancy and are yet to demonstrate a clear beneficial effect of vitamin D supplementation. There are a number of potentially interdependent mechanisms whereby vitamin D could dampen liver inflammation, by inhibiting hepatocyte apoptosis and liver fibrosis, modulating the gut microbiome and through altered production and transport of bile acids. While there has been a focus on vitamin D, other mediators induced by sun exposure, such as nitric oxide may also play important roles in curtailing liver inflammation.

## 1. What Is Non-Alcoholic Fatty Liver Disease?

Non-alcoholic fatty liver disease (NAFLD) is a spectrum of disorders ranging from simple steatosis to non-alcoholic steatohepatitis. NAFLD is linked with obesity, type-2 diabetes and metabolic syndrome, and is now the most prevalent liver disorder in western countries. It is caused by excessive accumulation of fat (triglycerides) in the liver [1] and results in abnormal lipid metabolism, but the underlying molecular mechanisms are under debate [2,3]. In the *double-hit* hypothesis, lipid accumulation in the liver is followed by the release of pro-inflammatory mediators that induce inflammation, hepatocyte death and fibrosis [4]. Others suggest that *free fatty acids* and cytokines (such as tumour necrosis factor-α, TNFα) are hepatotoxic and underpin the molecular pathogenesis of NAFLD [5]. A third hypothesises is that *multiple hits* are required, including genetic, environmental and nutritional factors, involving diverse parallel processes including fatty acids, bacterial lipopolysaccharides, cytokines, chemokines and adipokines, which acting in concert induce NAFLD [6,7]. Here we discuss the evidence for sunlight-derived mediators, such as vitamin D and nitric oxide (NO) in suppressing hepatic inflammation that contributes towards the severity of NAFLD.

## 2. Sunlight-Derived Ultraviolet Radiation—A Systemic Modulator of Inflammation

**Figure 1:**
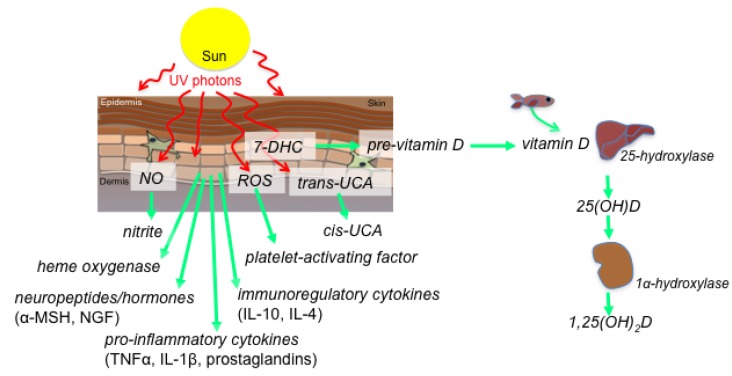
Skin exposure to ultraviolet (UV) photons from sunlight results in the synthesis of effector molecules that modulate immune and metabolic processes. These include: skin release of nitric oxide (NO), increasing serum levels of nitrite; initiation of the conversion of precursor molecules such as reactive oxygen species (ROS), *trans*-urocanic acid (UCA) and 7-dehydrocholesterol (7-DHC) into platelet-activating factor, *cis*-UCA and 1,25-dihydroxyvitamin D (1,25(OH)_2_D, calcitriol), respectively; and, the release of heme oxygenase, neuropeptides and neurohormones (e.g., α-melanocyte stimulating hormone (α-MSH), nerve growth factor (NGF)), immunoregulatory (interleukin(IL)s-4 and -10) and pro-inflammatory (tumor necrosis factor-α, IL-1α, prostaglandins) cytokines. Sun exposure of the skin results in systemic production of active vitamin D (1,25(OH)_2_D) through hydroxylation of vitamin D in the liver and 25-hydroxyvitamin D (25(OH)D) in the kidneys. These events can also occur locally in skin and immune cells that express the hydroxylating enzymes.

Ultraviolet radiation (UVR) derived from the sun has both local and systemic effects on health. Skin exposure to UVR induces several immune effector molecules, including vitamin D, NO, heme oxygenase, *cis*-urocanic acid and serotonin (Figure 1, reviewed in [8]). The UVR spectrum is divided into three distinct biologically active wavelength bands: UVA (315–400 nm), UVB (280–315 nm) and UVC (100–280 nm); and, since the latter does not penetrate the ozone layer of the atmosphere it will not be discussed here. UVB is largely absorbed in the epidermis while around 30% of UVA reaches the dermis. Here, we discuss the roles of vitamin D, NO and heme oxygenase as immune and inflammatory modulators induced by UVR.

Vitamin D_3_ is formed when 7-dehydrocholesterol in skin cells, predominantly keratinocytes, absorb UVB photons. Upon binding to vitamin D binding protein, vitamin D circulates to the liver for hydroxylation at the 25-position (to form 25(OH)D) and subsequently to the kidney for hydroxylation to form active, but short-lived, 1,25-dihydroxyvitamin D (1,25(OH)_2_D)(Figure 1). The half-life of 25(OH)D is 30–40 days so this is recognized as the storage circulating form. We have recently reviewed the effects of vitamin D on the immune system [9,10]. Most immune cells (and epithelial cells), express the inducible enzyme, CYP27B1 (1α-hydroxylase), so they too can make 1,25(OH)_2_D, and to high levels (up to 1 nM) for autocrine and paracrine effects at the local tissue level [11]. Innate immunity is our first line of defense against microbes, and macrophages and dendritic cells of the innate system can be activated through pathogen-recognition molecules (e.g., Toll-like receptors or NOD receptors) to increase CYP27B1 expression and thus, 1,25(OH)_2_D production. As all immune cells express the vitamin D receptor (VDR), 1,25(OH)_2_D may have autocrine effects to limit immune cell activation and inflammation. Other studies suggest that expression of the VDR is reduced by macrophage differentiation, providing a limit on the potential for these cells to respond to 1,25(OH)_2_D (reviewed by [12]). In the liver, VDR expression is mainly detected on non-hepatocytes, including sinusoidal epithelial cells, Kupffer cells, and particularly hepatic stellate cells, suggesting that they (and not hepatocytes) harbour the capacity to respond quickly to local synthesis of 1,25(OH)_2_D [13]. Similarly other components of the vitamin D unit (including enzymes that inactivate 1,25(OH)_2_D; e.g., CYP24A1) may be regulated in response to immune activation (reviewed in [9]). The adaptive immune system is also modulated by 1,25(OH)_2_D, by inhibiting the activation of dendritic cells, decreasing cytokine production and suppressed T cell activation, and promoting the development of regulatory T cells.

NO can be formed by two independent mechanisms: production from l-arginine via the NO synthase (NOS) family of enzymes (neuronal NOS or NOS-I, inducible NOS or NOS-II, and endothelial NOS or NOS-III), and the serial reduction of nitrate (NO_3_^−^) to nitrite (NO_2_^−^) and then to NO. These activities are carried out by a multitude of enzyme systems and are oxygen dependent, although while NOS requires oxygen to form NO, the nitrate reduction pathway is inhibited by its presence. In addition to these enzymatic formation routes NO can also be released from preformed storage forms by light. The mechanisms of release by skin exposure to UVA radiation are discussed below. UVA and UVB radiation induces NOS-II and III expression in murine skin [14,15]. NO constitutively produced from eNOS limits keratinocyte apoptosis in a cGMP-dependent manner, which may thus ‘harden’ the skin against premature apoptosis due to sun exposure. In humans, applying the NOS antagonist N(G)-monomethyl-l-arginine to the skin prevented UVA-induced immunosuppression to a recall antigen, and depletion of epidermal Langerhans cells, the antigen-presenting cells of the epidermis [15].

Heme oxygenase (HO)-1 is an enzyme that catalyses the degradation of heme, and is typically produced in response to oxidative stress. Heme degradation is associated with the formation of carbon monoxide (CO) and biliverdin; CO can inhibit the production of pro-inflammatory cytokines while biliverdin acts as an antioxidant [16]. HO-1 may play a cytoprotective role in the skin following exposure to sunlight. It is induced in skin by exposure to UVA radiation, where it acts as an antioxidant [17]. UVA-induced HO-1 may reduce the ability of UVB radiation to suppress immune responses through IL-6 and estrogen receptor-β-dependent mechanisms [18,19].

## 3. Hepatic Inflammation Linked with NAFLD

Chronic and/or excessive liver inflammation exacerbates the severity of NAFLD by killing hepatocytes [20]. Myofibroblasts replace dead hepatocytes, hindering liver function through fibrosis [21]. Hepatocyte death may be caused by profound mitochondrial dysfunction [22], the expression of extracellular signals that promote cell death (e.g., members of the TNF superfamily) and through internal responses that deal with stress, such as the ER stress response, autophagy and production of reactive oxygen or nitrogenous species (reviewed by [20]). Dying hepatocytes perpetuate the pro-inflammatory signal by activating innate immune cells through pattern recognition receptors [6,20,23]. Below we discuss briefly the roles of immune cells and central effector molecules that drive inflammation in the liver.

### 3.1. What Are the Roles of Resident and Infiltrating Immune Cells in Modulating Liver Inflammation?

A variety of immune cells contribute to hepatic inflammation, beginning with tissue resident Kupffer (resident macrophages) and natural killer cells, followed rapidly by innate cells (neutrophils, monocytes, inflammatory macrophages), and then finally by infiltrating natural killer T cells, B cells and T cells (reviewed by [6]). Kupffer cells are activated by pathogen- or damage-associated molecular patterns (PAMPs and DAMPs). These molecules are not usually expressed in the liver. During processes of sterile inflammation and tissue injury with progression to NAFLD, DAMPs are expressed/released by hepatocytes to activate receptors on Kupffer and other immune cells [23]. NAFLD is associated with changes to the gut microbiome, which can result in the release of PAMPs and damaging bile acids and toxins that promote inflammation in the gut and liver (reviewed in [3]). This *gut-liver axis* hypothesis suggests that PAMPs of intestinal origin and DAMPs of liver origin collectively drive hepatic liver inflammation and NAFLD [3,23]. In addition, inflammatory signals may arise from hypertrophic adipocytes and macrophages in adipose tissue [6]. There is overlap in the inflammatory pathways involving PAMPs and DAMPs; those important for the progression of NAFLD include those that involve the toll-like receptors, TLR4 and TLR9 [23]. The collective expression of PAMPs/DAMPs results in proinflammatory cytokine release (e.g., IL-1β, IL-6, IL-12, IL-18), the recruitment of immune cells and further hepatocyte apoptosis [6].

### 3.2. What Are the Central Effector Molecules that Drive Liver Inflammation?

A shift in the balance of the expression of anti-inflammatory cytokines (e.g., IL-10) towards pro-inflammatory cytokines (e.g., TNFα, IL-6) is required for the development of NAFLD [2]. Obesity induces TNFα production by adipocytes, facilitating insulin resistance in these cells and increased lipolysis. This increases the pool of free fatty acids circulating for uptake in the liver and combined with reduced clearance capacity by mitochondria results in the accumulation of triglyerceride droplets in hepatocytes (reviewed by [2]). Pro-inflammatory signals may be derived from liver cells or immune cells migrating into the liver and include cytokines, reactive oxygen and nitrogenous species, chemokines and lipid mediators [6,20]. Many of these mediators are released by keratinocytes or other skin cells following dermal exposure to UVR, including TNF [24], IL-6 [25], prostaglandins [26] and NO [27]. It is therefore counterintuitive that sunlight could prevent liver inflammation and NAFLD. However, there is accumulating evidence that skin exposure to sunlight-derived UVR has potent anti-inflammatory effects in the liver, which we detail below. 

## 4. Evidence for the Control of Hepatic Inflammation by Ultraviolet Radiation: Vitamin D-Dependent and Independent Pathways

### 4.1. Effects of Phototherapy on Severity of NAFLD

Phototherapy effectively suppresses the severity of NAFLD in rodent models, through a mechanism that is partially dependent on vitamin D [28,29]. Nakano *et al.* first demonstrated the efficacy of phototherapy to curtail the severity of NAFLD in male Lewis rats fed a choline-deficient and iron-supplemented L-amino acid (CDAA)-defined diet [28]. The phototherapy was administered for 12 h/day (unknown dose) from an artificial sunlight source (undefined light spectrum) [28]. It reduced hepatocyte apoptosis, inflammation, fibrosis (but not steatosis), insulin/leptin resistance, hepatic and circulating triglyceride levels, and the progression to non-alcoholic steatohepatitis (NASH). In addition, phototherapy suppressed liver levels of TNFα and TGFβ mRNA and α-smooth muscle actin protein [28]. The same phototherapy also reduced liver inflammation and fibrosis in male Zucker fa/fa rats (a leptin-deficient model of obesity) [28]. With skin exposure to UVR, circulating 25(OH)D and 1,25(OH)_2_D levels increased relative to the control treatment; however, the CDAA diet alone suppressed these levels to 20%–30% of their original concentrations. Supplementation of CDAA-fed rats with 1,25(OH)_2_D (0.4 μg/kg orally 3 times/week) reduced the severity of liver hepatitis, in a manner similar to phototherapy, suggesting that some of its effects were dependent on vitamin D [28].

Our studies suggest that UVR has the potential to suppress NAFLD development through vitamin D-dependent and -independent mechanisms. In C57Bl/6 male mice fed a high fat diet for 12 weeks, chronic sub-erythemal UVR (non-burning, low dose, 1 kJ/m^2^ UVB twice a week) or erythemal UVR (burning, high dose, 4 kJ/m^2^ UVB once a fortnight) suppressed liver steatosis and lobular ballooning [29]. There was no difference in the liver pathology observed with the different doses of UVR [29]. When male mice were fed a high fat diet (not supplemented with vitamin D), there was no increase in serum 25(OH)D levels with UVR [29], which may have been caused by diminished skin levels of 7-dehydrocholesterol in male mice [30]. Thus, UVR suppressed the severity of NAFLD in a manner that was independent of vitamin D. We compared the effects of UVR to dietary vitamin D, and found that while dietary vitamin D_3_ (~2000 IU/kg added to diet) did significantly alleviate liver steatosis and ballooning, UVR was more potent. We also observed significant reductions in circulating TNFα levels in mice supplemented with vitamin D [29], but not UVR, suggesting that dietary vitamin D and UVR may suppress NAFLD through differing yet complementary mechanisms. 

### 4.2. Associations between Vitamin D and NAFLD

The association between serum 25(OH)D concentrations and the presence and/or severity of NAFLD has been widely investigated in the last two years, building on a small number of studies published prior to 2013 (summarised in Table 1). The majority of these studies were in adults [31,32,33,34,35,36,37,38,39,40,41,42,43], with a few addressing associations in childhood and adolescence [44,45,46,47,48]. Most used ultrasound to detect NAFLD [31,32,35,36,37,38,39,41,42,44,47,48], with some relying on serum alanine transaminase (ALT) as a biomarker to detect ‘suspected NAFLD’ [40,45,46]. A few employed the gold-standard method of liver biopsy to conclusively diagnose NAFLD [33,34,43]. A wide range of ethnicities has been investigated, with studies originating from North America, Europe, Asia and Australia.

Overall, most observational studies support an inverse association between serum 25(OH)D concentrations and NAFLD [31,32,34,35,36,37,38,39,40,41,42,43,44,46,47,48], independent of potential confounders, such as adiposity and insulin resistance. We recently observed a significant inverse association between serum 25(OH)D concentrations and NAFLD in 17 year-old Western Australian adolescents participating in a population-based cohort (*n* = 718) [44]. A recent meta-analysis of 17 cross-sectional and case-control studies of serum 25(OH)D concentrations and NAFLD showed that, compared to controls, NAFLD patients were 1.26 times more likely to be vitamin D deficient [49].

The reliability of results from observational studies investigating the association between serum 25(OH)D concentrations and NAFLD may be affected by a number of factors, including the method used to detect NAFLD, the type of assay used to measure serum 25(OH)D concentrations, and the covariates included in the analyses. Although liver ultrasound provides a useful non-invasive estimate of histological hepatic steatosis in large-scale studies [50], it becomes less sensitive when detecting low levels of steatosis. Similarly, ALT concentrations are sometimes used as a biomarker to detect “suspected NAFLD”, but may be relatively insensitive and nonspecific for NAFLD [51,52].

Most observational studies have relied on immunoassay methods, which are highly variable in the measurement of serum 25(OH)D concentrations [53,54], and none have reported using an assay that is certified to the standard reference method developed by the National Institute of Standards and Technology and Ghent University [55]. Not all studies have adequately adjusted for adiposity or season of blood collection, and the sample size of many studies has been small and limited to patients at specific clinical centres. Importantly, evidence to date is limited to cross-sectional analyses, and the possibility of reverse causality cannot be ruled out. Ultimately, although an inverse association between serum 25(OH)D concentrations and NAFLD is widely reported in the current literature, there is no definitive evidence from human studies that supports a causal association between low serum 25(OH)D concentrations and the development or severity of NAFLD.

**Table 1:** Associations of circulating 25(OH)D and NAFLD.

Reference	Location	Sample Size	Percent Male	Age	NAFLD Detection	Assay Method	Significant Association	Summary of Findings
Barchetta *et al.*, 2011 [31]	Italy	262	53	Adults	Ultrasound	DiaSorin LIAISON	Inverse	Low 25(OH)D was associated with NAFLD independent of metabolic syndrome, diabetes and insulin-resistance profile
Bril *et al.*, 2014 [33]	USA	239	85	18–70 year	Biopsy	DiaSorin LIAISON	None	Plasma 25(OH)D was not associated with insulin resistance, liver fat accumulation or severity of NASH
Dasarathy *et al.*, 2014 [34]	USA	187	27	Adults	Biopsy	Standard automatic colorimetric methods	Inverse	Plasma 25(OHD was an independent predictor of NAFLD activity score
Hao *et al.*, 2014 [35]	China	514	100	Adults	Ultrasound	Electrochemiluminescence immunoassay (Roche)	Inverse	Serum 25(OH)D was inversely associated with NAFLD, even in subjects with normal total body fat
Jablonski *et al.*, 2013 [36]	USA	1214	26	≥18 year	Ultrasound	RIA	Inverse	Compared with matched controls, NAFLD patients had significantly lower serum 25(OH)D
Kasapoglu *et al.*, 2013 [37]	Turkey	613	21	Adults	Ultrasound	Not reported	Inverse	Low 25(OH)D was associated with NAFLD among non-obese subjects
Kucukazman *et al.*, 2014 [38]	Turkey	211	44	Adults	Ultrasound	RIA	Inverse	Serum 25(OH)D was lower in patients with NAFLD compared to those without NAFLD
Li *et al.*, 2013 [39]	China	1248	55	≥20 year	Ultrasound	DiaSorin RIA	None	Serum 25(OH)D was not significantly associated with the presence of NAFLD
Liangpunsakul *et al.*, 2011 [40]	USA	1287	50	≥20 year	Alanine transaminase	RIA	Inverse	Significant inverse association between serum 25(OH)D and unexplained elevation in alanine aminotransaminase
Rhee *et al.*, 2013 [41]	Korea	6567	100	24–75 year	Ultrasound	Electrochemiluminescence immunoassay (Roche)	Inverse	Reduced risk for NAFLD with higher serum 25(OH)D, independent of obesity and metabolic syndrome
Seo *et al.*, 2013 [42]	Korea	1081	32	40–69 year	Computed tomography	DiaSorin LIAISON	Inverse	In subjects with diabetes or insulin resistance, low vitamin D status was associated with NAFLD, independent of visceral obesity
Targher *et al.*, 2007 [43]	Italy	120	67	Adults	Biopsy	DiaSorin LIAISON	Inverse	Compared with matched controls, NAFLD patients had lower serum 25(OH)D
Bhatt *et al.*, 2013 [32]	India	335	71	Adults	Ultrasound	DiaSorin RIA	Inverse	Low serum 25(OH)D was independently associated with NAFLD
Black *et al.*, 2014 [44]	Australia	994	52	17 year	Ultrasound	LC-MS/MS	Inverse	Lower serum 25(OH)D was significantly associated with NAFLD, independent of adiposity and insulin resistance
Katz *et al.*, 2010 [45]	USA	1630	52	12–19 year	Alanine transaminase	DiaSorin RIA	None	No independent association between vitamin D status and NAFLD after adjusting for obesity
Malespin *et al.*, 2014 [46]	USA (Chinese American)	407	51	6–18 year	Alanine transaminase	Immunochemiluminometric assay	Inverse	Suspected NAFLD was associated with lower 25(OH)D after adjusting for BMI, sex, triglycerides, total cholesterol, LDL, HDL
Nobili *et al.*, 2014 [47]	Italy	73	64	10–15 year	Ultrasound	HPLC	Inverse	25(OH)D was inversely associated with NASH and fibrosis in overweight and obese children with NAFLD
Pirgon *et al.*, 2013 [48]	Turkey	87	48	11–15 year	Ultrasound	DiaSorin LIAISON	Inverse	In obese adolescents, those with NAFLD had significantly lower 25(OH)D than those without NAFLD

An unexplored possibility, which deserves further consideration, is that circulating vitamin D levels may represent a proxy for bodily exposure to sunlight [56], a view consistent with the notion that individuals with lower 25(OH)D concentrations are more susceptible to disease development. Another explanation for the negative associations of vitamin D and NAFLD could relate to an impairment of liver function, where inflammation and the disease process itself might curtail the activity of 25-hydroxylases expressed by hepatocytes, preventing conversion of vitamin D into 25(OH)D. In a rodent model of liver inflammation, ligation of bile ducts induced cholestasis, inflammation and fibrosis in the liver, and inhibited the activity of vitamin D 25-hydroxlase [57]. Furthermore, expression of the 25-hydroxylase, CYP2R1 was inversely related to lobular inflammation in patients with NASH [58]. These studies suggest that hepatic inflammatory processes may hinder circulating 25(OH)D by affecting the hydroxylation of vitamin D in the liver.

The influence of vitamin D on the development and progression of NAFLD may vary depending on the presence of certain genetic polymorphisms. Indeed, genetic variations in vitamin D metabolism have been identified and may be associated with liver fibrosis and stiffness [59]. VDR expression in the liver and inflammatory cells of chronic liver disease patients is negatively associated with the severity of liver histology in both NASH and hepatitis C patients [58]. VDR expression on cholangiocytes was inversely correlated with steatosis severity, lobular inflammation and NAFLD score, and expression of CYP2R1 in hepatocytes of NASH patients correlated strongly with VDR positivity on liver inflammatory cells. Adams and colleagues previously observed that a common single nucleotide polymorphism (rs222054) in the group specific component gene, which encodes vitamin D binding protein, was associated with NAFLD [60]. Based on these current findings, the genetic variation in vitamin D metabolism deserves further research with respect to NAFLD.

### 4.3. Vitamin D Supplementation and NAFLD

A number of open clinical trials investigating the effects of vitamin D supplementation on NAFLD are currently underway, including three listed at *www.clinicaltrials.gov* [61] (using vitamin D and non-alcoholic fatty liver disease as keywords). One trial will examine the effects of vitamin D_3_ (2100 IU/day orally) compared to placebo on serum ALT in patients with NASH over 48 weeks (NCT01571063). In another, the capacity of vitamin D_3_ (800 IU/day orally) combined with Docosahexaenoic Acid (500 mg/day orally) to affect NAFLD activity is to be compared with placebo in a pediatric population for 12 months (NCT02098317). A final trial is determining the efficacy of vitamin D_2_ (50,000 IU/week for 6 weeks, and then bi-weekly for 6 months orally) compared to placebo to affect hepatic triglyceride content in children and adult patients with type-2 diabetes (NCT02132442). All three have estimated enrolments of <70.

To our knowledge, there is only one published study investigating the effect of vitamin D supplementation in patients with NAFLD. Sharifi and colleagues [62] investigated the effect of vitamin D supplementation on serum aminotransferases, insulin resistance, oxidative stress and inflammatory biomarkers in adult patients with NAFLD in a parallel, double-blind, placebo-controlled study based in Iran. Patients were supplemented with 50,000 IU (1250 µg) of vitamin D_3_ (*n* = 27) or a placebo (*n* = 26) every 14 days for four months. After adjusting for baseline covariates, including season and body fat percentage, vitamin D supplementation improved serum high-sensitive C-reactive protein and serum malondialdehyde concentrations. There was no significant difference between groups in changes in serum ALT, aspartate transaminase, alkaline phosphatase and insulin resistance. 

Although the results of Sharifi *et al.* are relatively unsupportive of a benefit of vitamin D supplementation in adults with NAFLD, the study group was small and the intervention limited to four months [62]. All of the above-mentioned ongoing clinical trials are aiming to recruit similar or smaller group sizes but will test the effects of vitamin D for longer (≥6 months). Furthermore, it will be important to compare the clinical effectiveness of vitamin D supplementation with other successful strategies. Indeed there is good quality evidence to support the beneficial effect of lifestyle changes that promote weight loss (including exercise and diet changes) in reducing the risk of NAFLD [63], and weight loss has been shown to improve the histological features of NASH [64]. Interestingly, exercise alone can have positive effects on NAFLD independently of weight loss [65]. As much exercise is performed outdoors, we suggest that some its benefits may be due to increased sun exposure and the potential bioactivity of vitamin D and/or other UV-induced mediators.

### 4.4. Mechanisms by Which Vitamin D Could Suppress Liver Pathology

Vitamin D may prevent liver pathology and the development of NAFLD through the suppression of related and potentially interacting pathways that involve hepatocyte apoptosis, liver inflammation and fibrosis, oxidative stress, the expression of protective adipokines, and changes to the composition of the gut microbiome.

#### 4.4.1. Vitamin D Inhibits Hepatocyte Apoptosis

Feeding rats calcitriol (1,25(OH)_2_D; 1 μg/kg/day, intraperitoneal injection) suppressed the acute rejection of liver allografts [66,67]. Vitamin D may have contributed towards allograft survival by inhibiting hepatocyte apoptosis, as suggested by its ability to regulate the expression of apoptosis-associated proteins in the liver, increasing anti-apoptotic Bcl-2 and Bcl-xL proteins, and decreasing pro-apoptotic Bax and Caspase-3 proteins [66]. Calcitriol also inhibited expression of FasL [66], a protein important for the ability of cytotoxic T cells to target foreign cells.

#### 4.4.2. Vitamin D Reduces Liver Fibrosis

Hepatic stellate cells secrete extracellular matrix, which serves as a scaffold for cellular reconstitution and the formation of fibrotic tissue. Active 1,25(OH)_2_D can directly repress the ability of these cells to form type I collagen [68]. When hepatic stellate cells were obtained from morbidly obese patients with biopsy-proven NAFLD, liver fibrosis was associated with increased fragmentation of the VDR protein [69]. *In vitro* treatment with 1 μM vitamin D_2_ suppressed the pro-fibrogenic activity of TGFβ from hepatic stellate cells, modifying the expression of the SMAD2 protein [69]. Other studies suggest that vitamin D may slow the proliferation of hepatic stellate cells (reviewed by [70]). Together, these observations indicate that vitamin D may affect liver fibrosis controlled by hepatic stellate cells through multiple mechanisms.

#### 4.4.3. Does Vitamin D Modulate Adipokine Expression?

Adiponectin is an adipokine (secreted from adipose tissue), which regulates glucose and fatty acid oxidation, but may play a protective role in metabolic processes. Adiponectin may suppress liver fibrosis by inhibiting secretion of TNFα by hepatic stellate cells [71]. Some studies suggest a positive relationship between serum levels of adiponectin and 25(OH)D; however, not all support this link (reviewed in [72]). There is also no agreement in results derived from preclinical studies. Phototherapy increased circulating adiponectin and 25(OH)D levels in CDAA-fed Lewis rats [28], but dietary vitamin D had no effect on adiponectin levels in Sprague-dawley rats fed a ‘westernised diet’ that was high in fat and fructose [73] or in mice fed a high fat diet [29]. This might suggest a factor other than vitamin D being released by UVR that reduced plasma adipokine levels. Other adipokines, which may contribute towards the progression of NAFLD include resistin and leptin [71]. However, like adiponectin, there is insufficient evidence, from either preclinical [73,74] or human [75,76,77,78] studies to conclusively link their expression with the protective effects of vitamin D.

#### 4.4.4. Vitamin D Alters the Gut Microbiome and Production of Bile Acids

As highlighted above, PAMPS derived from the gut may contribute towards liver inflammation through the *liver-gut axis*. Roth *et al* [73] hypothesised that in the absence of vitamin D the gut microbiome may change, resulting in enhanced endotoxin exposure and TLR activation, with increased expression of TLR-2, -4 and -9 mRNAs observed in the livers of vitamin D-deficient rats fed a westernised diet [73]. As we have recently reviewed, vitamin D may induce antimicrobials and promote immune tolerance to change the gut microbiome [9]. Other important pathways regulated by vitamin D include the metabolism of bile acids by gut microflora and hepatocytes. Bile acids are produced by hepatocytes and stored in the gallbladder before release into the gut for fat digestion [3]. Bile acids share structural similarity with vitamin D and regulate energy metabolism by interacting with bile acid receptors. Loss in the expression of these receptors has been linked with development of NAFLD and liver carcinogenesis (reviewed in [3]). Vitamin D may directly suppress the synthesis of bile acids by hepatocytes (review in [79]). In addition, changes to the gut microbiome driven by vitamin D deficiency may alter the levels or type of bile acids present in the gut. Indeed, the hepatotoxic bile acid, lithochloric acid [3], interacts with the vitamin D receptor (VDR), which acts as bile acid receptor and targets the acid for degradation [80]. Activation of the VDR by other bile acids or active 1,25(OH)_2_D [80] may enable the degradation of toxic bile acids in the gut and protect from subsequent liver inflammation. 

#### 4.4.5. Vitamin D Suppresses Proinflammatory Cytokines and Mediators of Oxidative Stress

Increased lobular inflammation and expression of mRNAs encoding the proinflammatory cytokines IL-1β, IL-4 and IL-6 was detected in the livers of vitamin D-deficient Sprague-Dawley male rats fed a westernised diet [73]. The increased proinflammatory cytokine expression was linked with TLR activation, which as detailed above may have reflected changes in the gut microbiome and the release of PAMPS. Proinflammatory oxidative stress pathways in the liver may also be regulated by vitamin D. Roth *et al.* observed enhanced expression of heme oxygenase-1 mRNA in the liver of vitamin D-deficient rats fed the westernised diet [73]. Orally administered vitamin D_3_ (12 μg/kg body weight for 2 weeks) increased the gene expression of antioxidant defence molecules such as glutathione peroxidase and superoxide dismutase in the livers of streptozotocin-induced diabetic rats [81]. In the liver, active 1,25(OH)_2_D may directly suppress the synthesis of proinflammatory cytokines and oxidative stress molecules.

**Figure 2:**
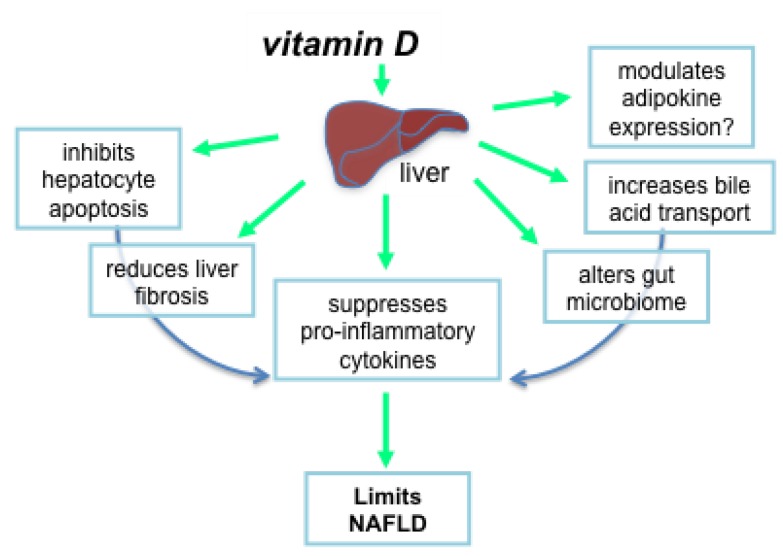
Vitamin D may limit the progression of non-alcoholic fatty liver disease through multiple, potentially interacting mechanisms.

Vitamin D may also regulate inflammation by affecting bile acid transport in the liver. In vitamin D deficient mice, the expression of the ileal apical sodium-dependent bile acid cotransporter was reduced in the livers of mice with more severe hepatic steatosis and inflammation induced by feeding mice a high fat diet [82]. Supplementation of these mice with intramuscular 1,25(OH)_2_D (5 ng/g body weight, twice a week) reversed the effects of vitamin D deficiency on the expression of this bile acid transporter and the extent of liver inflammation. In addition, there are various indirect mechanisms, such as hepatocyte apoptosis and effects on the gut microbiome, whereby vitamin D targets inflammation (see Figure 2).

### 4.5. Other UVR-Induced Mediators That Could Suppress Liver Inflammation

Exposure of skin to UVR induces a suite of mediators, many of which could curtail the severity of NAFLD; here, we focus on the potential protective effects of UV-induced NO and heme oxygenase.

#### 4.5.1. Activation of Dermal Nitric Oxide (NO) by UVR

NO is an important vasodilator and signaling molecule within the cardiovascular, pulmonary, neuronal and immune systems [83]. However, the role that NO plays in modulating liver inflammation and hepatotoxicity is incompletely understood (reviewed in [84,85,86,87]). Factors that may determine whether NO is hepatotoxic or hepatoprotective are how, where and for how long it is produced in the liver. NO synthases convert intracellular l-arginine into citrulline and NO. Expression of endothelial (e)NOS is constitutive and has beneficial effect on the vasculature, while inducible (i)NOS is generated by stress and results in sustained NO production at much higher levels, inducing nitrosative stress (reviewed in [20]). Of relevance here is the ability of 1,25(OH)_2_D to regulate NO synthesis. Treatment of human endothelial cells with 1,25(OH)_2_D increased the expression of NO through activation of eNOS [88], and VDR mutant mice have a lower bioavailability of NO due to reduced eNOS expression in their vasculature, leading to endothelial dysfunction and increased arterial stiffness [87]. In contrast, 1,25(OH)_2_D inhibited iNOS expression and reduced NO production by LPS-stimulated macrophages (RAW264.7 cells) at physiologically-relevant doses [89]. Similarly, 1,25(OH)_2_D administration selectively inhibited expression of the inducible cyclooxygenase isoform, COX-2, an enzyme implicated in inflammation, in cultured macrophages [90] and prevented symptoms of allergic asthma by suppressing iNOS in rats [91]. These studies suggest that UV-induced 1,25(OH)_2_D might promote the expression of eNOS by endothelial cells to increase constitutive expression of NO, protecting hepatocytes from nitrative/oxidative injuries caused by a NO burst invoked by iNOS. Further chemical reactions of NO with superoxide form peroxynitrite (ONOO^−^), and reduce inflammation by downregulating COX-2 expression. In addition, an alternate pathway (known as the nitrate-nitrite-nitric oxide pathway) for NO production exists, where the anions nitrate and nitrite are reduced back to NO [92]. The relative contribution of these pathways to the formation of dermal NO storage forms [27] and hepatic protection is currently unclear.

Skin exposure to UVR mobilizes NO bioactivity from skin, increasing serum nitrite levels within 20 min of skin exposure, with downstream beneficial suppressive effects on blood pressure [93]. These effects were independent of NOS, suggesting an important role for pre-formed cutaneous stores of NO, which were detected in the upper layers of the epidermis [93]. Similarly, we observed increased levels of NO in the dermis of mice exposed to UVR, and the NO donor, SNAP [29]. In mice fed a high fat diet, the extent of liver steatosis and lobular ballooning was significantly reduced by twice-weekly treatment with the NO donor, SNAP, or low-dose irradiation with UVR (1 kJ/m^2^ UVB), with the effects of UVR reversed by the NO scavenger cPTIO [29]. The mild blood pressure lowering effects of UVR might be explained by the dilation of small blood vessels in the skin secondary to the release of a mediator such as NO acting in an autocrine fashion. In contrast, hepatic protection by UVR would demand the transduction of bioactivity from the skin to the circulation with subsequent transport to the target organ in a paracrine fashion. The precise mechanism by which UVR-induced mobilization of NO bioactivity reduces the severity of NAFLD is unknown; however, one means could be through regulation of hepatocyte viability. Alternatively, NO may act as an antioxidant [94], leading to the inhibition of reactive oxidant species induced lipid oxidation. NO is also known to modulate mitochondrial function and biogenesis [95], and as already discussed, hepatocyte death may be promoted by mitochondrial dysfunction [22], but further studies are necessary.

#### 4.5.2. NO-Mediated Upregulation of Heme Oxygenase Expression

There is uncertainty about the role of HO-1 in the liver and how its expression may affect the severity of NAFLD. Hepatic HO-1 protein levels are increased in the liver in patients with NASH relative to steatosis-positive controls [96], and there are reports of increased HO-1 mRNA, protein and/or activity in the livers of rodents with NAFLD [74,97,98]; yet other preclinical studies do not support these findings [99,100,101]. Interestingly, exposure of skin to UVA radiation increased activity of HO-1 in both the skin and liver [102], perhaps indicating a hepato-protective effect for UV-induced HO and control of NAFLD. Indeed, feeding Zucker-Diabetic-Fatty rats heme (twice weekly, 30 mg/kg intraperitoneally) upregulated HO-1 activity in the liver, attenuated hepatic inflammatory cytokine expression, hepatocyte ballooning and fibrosis [103]. As detailed above, our studies demonstrate that NO is released from dermal storage forms by UVR [94]. NO may increase HO-1 expression in a variety of tissues ([104]), There may be a role for NO-induced HO-1 in the development of NAFLD, but further mechanistic studies are required.

## 5. Conclusions

Here we review how chronic skin exposure to sunlight-derived UVR may control the development of NAFLD. In addition to vitamin D, UVR induces the production of mediators such as NO and HO-1, which may contribute towards the protective effects of sunlight through a variety of mechanisms that suppress liver inflammation. Chronic skin exposure to UVR may halt the development of NAFLD [28,29], but we do not know whether it is possible to intervene; that is, can UVR or its mediators be used to treat already established NAFLD? Additional studies are required to test whether the observed associations of increased circulating 25(OH)D and positive pre-clinical findings for reduced NAFLD incidence/severity can be translated into recommendations for vitamin D supplementation in the clinic.
